# Supplementary material for: Empirical methods for controlling false positives and estimating confidence in ChIP-Seq peaks
Source: BMC Bioinformatics. 2008 Dec 5;9:523. doi: 10.1186/1471-2105-9-523 (PMC2628906; doi:10.1186/1471-2105-9-523)
Supplement: Additional File 1 — A variety of html documents from the USeq web site detailing the available applications, their best usage, output file type descriptions, command line menus, etc. [file 1471-2105-9-523-S1.zip › USeqUserGuides/JavaDoc.zip!/JavaDoc/bioroot/Util.html]

Util


|  |  |  |
| --- | --- | --- |
| **Overview** \| **Package** \| **Class** \| **Use** \| **Tree** \| **Deprecated** \| **Index** \| **Help** | |  |
| **PREV CLASS**   NEXT CLASS | **FRAMES**    **NO FRAMES**     **All Classes** |  |
| SUMMARY: NESTED \| FIELD \| CONSTR \| METHOD | DETAIL: FIELD \| CONSTR \| METHOD |  |


---


## bioroot Class Util

```
java.lang.Object
  bioroot.Util
```

---

``` public class Util extends java.lang.Object ```

Utility class for BioRoot

---

| **Field Summary** | |
| --- | --- |
| `static java.lang.String` | `contactEmail`             Who do you call? |
| `static int` | `demoUserId` |
| `static java.lang.String` | `filepath` |
| `static java.lang.String` | `fullPathToBlast` |
| `static java.lang.String` | `HTMLPath` |
| `static java.lang.String` | `IPPort` |
| `static int` | `maxFileSize`             Maximum allowable file size for upload |
| `static java.lang.String` | `SMTPAccount`             SMTP account to use for emailing |
| `static java.lang.String` | `smtpHostName` |
| `static java.lang.String` | `smtpPassword` |
| `static java.lang.String` | `smtpUser` |


**Constructor Summary**

`Util()`


| **Method Summary** | |
| --- | --- |
| `static java.lang.String` | `buildHTMLTableRows(java.lang.String[] ids, java.lang.String[][] columnRows, boolean checked)`             Build HTML table rows. |
| `static java.lang.String` | `fetchTableColumnRow(Preference pref, java.lang.String reagentType)`             Returns a table header row using the Preferece obj and a reagentType: Oligo, Plasmid, Strain... |
| `static UserBean` | `fetchUserBean(javax.servlet.http.HttpServletRequest request, javax.servlet.http.HttpServletResponse response)`             Sets a guest user bean if there is none. |
| `static java.lang.String` | `getViewLine(ReagentBean[] reagentBeans, int[] inView, Preference reagentPreference, java.lang.String reagentType)`             Returns hot linked html for 1-30 31-60 ... |
| `static java.lang.String[]` | `getVisibilities(UserBean userBean, DBUtil bioRoot)`             If user is part of an organization, it returns WWW, orgName, Lab Mates, Private. |
| `static java.lang.String` | `makeEditHistory(java.lang.String firstName, java.lang.String lastName, java.lang.String pastEditHistory)`             Makes an edit history entry. |
| `static Feature[]` | `makeFeatures(java.lang.String[] stringFeatures)`             Use to make a default Feature[]. |
| `static int[]` | `parseView(javax.servlet.http.HttpServletRequest request)`             Parses a 12-49 type parameter named view to just the ints |
| `static java.lang.String[]` | `splitConcat(java.lang.String concat)`             Safely splits a three item, colon delimited concat (ie 3: zyx:4-99: 235, 3999) into a String[3], OK to have internal colons provided no space follows the colon. |

**Methods inherited from class java.lang.Object**

`equals, getClass, hashCode, notify, notifyAll, toString, wait, wait, wait`

**Field Detail**

### IPPort

```
public static final java.lang.String IPPort
```

**See Also:**: Constant Field Values

---


### fullPathToBlast

```
public static final java.lang.String fullPathToBlast
```

**See Also:**: Constant Field Values

---


### filepath

```
public static final java.lang.String filepath
```

**See Also:**: Constant Field Values

---


### demoUserId

```
public static final int demoUserId
```

**See Also:**: Constant Field Values

---


### HTMLPath

```
public static final java.lang.String HTMLPath
```

**See Also:**: Constant Field Values

---


### smtpHostName

```
public static final java.lang.String smtpHostName
```

**See Also:**: Constant Field Values

---


### smtpUser

```
public static final java.lang.String smtpUser
```

**See Also:**: Constant Field Values

---


### smtpPassword

```
public static final java.lang.String smtpPassword
```

**See Also:**: Constant Field Values

---


### contactEmail

```
public static final java.lang.String contactEmail
```

:   Who do you call?

    **See Also:**: Constant Field Values

---


### SMTPAccount

```
public static final java.lang.String SMTPAccount
```

:   SMTP account to use for emailing

    **See Also:**: Constant Field Values

---


### maxFileSize

```
public static final int maxFileSize
```

:   Maximum allowable file size for upload

    **See Also:**: Constant Field Values


**Constructor Detail**

### Util

```
public Util()
```


**Method Detail**

### fetchUserBean

```
public static UserBean fetchUserBean(javax.servlet.http.HttpServletRequest request,
                                     javax.servlet.http.HttpServletResponse response)
```

:   Sets a guest user bean if there is none.

---


### getVisibilities

```
public static java.lang.String[] getVisibilities(UserBean userBean,
                                                 DBUtil bioRoot)
```

:   If user is part of an organization, it returns WWW, orgName, Lab Mates, Private.
    Otherwise it just returns the standard three.

---


### parseView

```
public static int[] parseView(javax.servlet.http.HttpServletRequest request)
```

:   Parses a 12-49 type parameter named view to just the ints

---


### getViewLine

```
public static java.lang.String getViewLine(ReagentBean[] reagentBeans,
                                           int[] inView,
                                           Preference reagentPreference,
                                           java.lang.String reagentType)
```

:   Returns hot linked html for 1-30 31-60 ... sets of reagent beans
    That in view is left to be body font size others are 
    Reaagent type = Oligo, Plasmid, Strain; needed for setting hot link.

---


### makeFeatures

```
public static Feature[] makeFeatures(java.lang.String[] stringFeatures)
```

:   Use to make a default Feature[]. Name: ColumnName (or none): order: visibility

---


### fetchTableColumnRow

```
public static java.lang.String fetchTableColumnRow(Preference pref,
                                                   java.lang.String reagentType)
```

:   Returns a table header row using the Preferece obj and a reagentType: Oligo, Plasmid, Strain...

---


### buildHTMLTableRows

```
public static java.lang.String buildHTMLTableRows(java.lang.String[] ids,
                                                  java.lang.String[][] columnRows,
                                                  boolean checked)
```

:   Build HTML table rows.

---


### splitConcat

```
public static java.lang.String[] splitConcat(java.lang.String concat)
```

:   Safely splits a three item, colon delimited concat (ie 3: zyx:4-99: 235, 3999) into a String[3],
    OK to have internal colons provided no space follows the colon.

---


### makeEditHistory

```
public static java.lang.String makeEditHistory(java.lang.String firstName,
                                               java.lang.String lastName,
                                               java.lang.String pastEditHistory)
```

:   Makes an edit history entry. firstName lastName (date). If this is a new entry set past EditHistory to null or "".


---


|  |  |  |
| --- | --- | --- |
| **Overview** \| **Package** \| **Class** \| **Use** \| **Tree** \| **Deprecated** \| **Index** \| **Help** | |  |
| **PREV CLASS**   NEXT CLASS | **FRAMES**    **NO FRAMES**     **All Classes** |  |
| SUMMARY: NESTED \| FIELD \| CONSTR \| METHOD | DETAIL: FIELD \| CONSTR \| METHOD |  |


---
